# The Establishment of Ultrasonic-Assisted Extraction for the Recovery of Phenolic Compounds and Evaluation of Their Antioxidant Activity from *Morus alba* Leaves

**DOI:** 10.3390/foods11030314

**Published:** 2022-01-24

**Authors:** Beatriz Martín-García, María José Aznar-Ramos, Vito Verardo, Ana María Gómez-Caravaca

**Affiliations:** 1 Department of Nutrition and Food Science, Campus of Cartuja s/n, University of Granada, 18071 Granada, Spain; bea91mg@ugr.es (B.M.-G.); mariajoseaznar@ugr.es (M.J.A.-R.); 2 Institute of Nutrition and Food Technology ‘José Mataix’, Biomedical Research Center, University of Granada, Avda del Conocimiento sn., 18100 Granada, Spain; anagomez@ugr.es; 3 Department of Analytical Chemistry, Faculty of Sciences, University of Granada, Avd. Fuentenueva s/n, 18071 Granada, Spain

**Keywords:** *Morus alba* leaves, phenolic compounds, Box-Behnken, HPLC-MS, antioxidant activity

## Abstract

Phenolic compounds of *Morus alba* leaves are bioactive compounds with beneficial properties for human health. Therefore, in this study, an optimization of ultrasonic assisted extraction by Box–Behnken design was used for the first time to optimize factors such as the percentage of ethanol, ratio solvent/sample (*v*/*w*) and extraction time to reach the highest phenolic compound amounts (evaluated by HPLC-MS) while also evaluating in vitro antioxidant activity using DPPH, ABTS and FRAP assays. The optimal extraction conditions were 40% ethanol, 1/400 (*w*/*v*) and 35 min. Applying these optimal conditions, which were identified and quantified by HPLC-MS, resulted in the extraction of 21 phenolic compounds. According to these results, the main phenolic compounds in *Morus alba* leaves are the phenolic glycoside and phenolic acid named protocatechuic acid-glucoside and caffeoylquinic. In addition, *Morus alba* leaf extract contains flavonols such quercetin-3-O-6-acetylglucoside and rutin, which represent more than 7% of its total phenolic content.

## 1. Introduction

Mulberry (*Morus alba* L.) is a plant from the family of *Moraceae* that is native to Asia and is usually employed as food for silkworm breeding. It is cultivated in subtropical and tropical regions and the plant adapts to different pedo-climatic conditions [1]. Mulberry leaves are rich in several bioactive compounds such as phenolic compounds, alkaloids, polysaccharides and vitamins [2]. Mulberry leaves contain calcium, carbohydrates, iron, proteins, vitamin B1, vitamin D and β-carotene, which are also considered to be a nutritious and could be used as foods or for their medicinal activity [3,4]. Mulberry leaves also contain phenolic compounds including flavonols such as rutin and other quercetin derivatives which all possess anti-diabetic, hypolipidemic, antihypertensive, anti-atherosclerotic and anticonvulsant properties [2,5,6]. These beneficial effects are related in part to the antioxidant activity of these phenolic compounds [3,7]. 

The extraction technique is highly important when it comes to the quantity of phenolic compounds obtained before analysis. It depends on several factors such as the solvent composition, the structure of the matrix and the technique used for the extraction [8]. Conventional extraction techniques in plant materials such as heating, refluxing or using Soxhlet apparatus could activate oxidation or hydrolysis of phenolic compounds, while maceration and percolation require longer extracting time. The recent development of new automated high throughput extractors such as microwave-assisted extraction (MAE), ultrasonic-assisted extraction (UAE) and pressurized-assisted extraction (PLE), have resulted in increased attention as these techniques have shown to be efficient in the recovery of bioactive phytochemicals [9]. However, MAE and PLE require investment in high cost instruments [10]. The advantages of ultrasonic-assisted extraction are simple, easy to handle and inexpensive compared with the others. In ultrasonic-assisted extraction, a shear force is produced by ultrasonic cavitation which breaks the plant cell wall, which in turn accelerates the transfer of bioactive compounds so as to extract solvent in shorter time than maceration or percolation [9,11]. There are previous studies about the extraction of phenolic compounds in *Morus alba* leaves by using ultrasonic-assisted extraction [12,13]. Nevertheless, there is very little information about the optimization of extraction conditions in *Morus alba* leaves in the previous analyses. In addition, a wide variation in phenolic recovery in plants has been reported by using different extraction conditions. With ultrasonic-assisted extraction, the most influential factors on the yield of phenolic compounds are the solid/liquid ratio, extraction time and solvent concentration [14]. Therefore, in this paper an optimization of extraction conditions by ultrasonic-assisted extraction bath was proposed in order to obtain the highest phenolic content and the highest antioxidant activity by DPPH, ABTS and FRAP assays in *Morus alba* leaf extracts. This extract obtained under optimal conditions, was characterized by using HPLC-MS. 

## 2. Materials and Methods

### 2.1. Samples

Samples were collected from Granada (Spain). Two cultivars of *Morus alba* leaves were collected from two different fields in Granada (Spain). Forty leaves from 3 different trees were picked-up in two different fields. The leaves were air dried in dark conditions at room temperature and they were milled using a 10 basic miller (IKA, Staufen, Germany) and they were sieved to obtain a particle size of 0.2 mm.

### 2.2. Chemicals 

All solvents were purchased from Merck KGaA (Darm-stadt, Germany), whereas water was obtained in situ using a Milli-Q system (Millipore, Bedford, MA, USA). Chemical standards of the phenolic compounds were acquired from Sigma-Aldrich (St. Louis, MO, USA).

### 2.3. Experimental Design

A Box–Behnken design (BBD) is more efficient than other experimental designs such as central composite design and the three-level full factorial designs where the efficiency of one experimental design is the number of coefficients in the estimated model divided by the number of experiments. In addition, BBD does not contain combinations for which all parameters are simultaneously at their highest or lowest levels. Therefore, these designs avoid unsatisfactory results, which occur when the experiments are performed under extreme conditions [15]. For all these reasons, in this study the optimization of the ultrasonic-assisted extraction to obtain the maximum phenolic recovery in *Morus alba* leaves was obtained with a BBD with 3 independent factors (X_1_: ethanol/water ratio (*v*/*v*), X_2_: solvent/sample ratio (*v*/*w*) and X_3_: extraction time (min) with 3 levels for each variable. The dependent variables (Y) were the sum of phenolic compounds (SPC) determined by HPLC-MS, and the antioxidant capacity obtained by DPPH, ABTS and FRAP assays (Table 1). Food and Drug Administration (FDA) has labeled ethanol as a generally recognized safe solvent to use in food products, for this reason this solvent was chosen for the extraction of phenolic compounds [16]. The percentage of ethanol/water was 0–100% (*v*/*v*), the solid-to-solvent ratio was from 1/20 to 1/500 (*w*/*v*) and the extraction time was from 10 min to 90 min and, these parameters were chosen based on the extraction conditions employed by previous studies for the recovery of phenolic compounds in *Morus alba* leaves [12,17,18]. The design comprised 15 experiments with 3 center points (Table 1).

Response surface methodology (RSM) is the most relevant multivariate technique employed in analytical optimization. The relationships between the response and independent variables is described as a second-order polynomial equation. The data were processed with the statistical software STATISTICA 7.0 (2002, StatSoft, Tulsa, OK, USA).

### 2.4. Ultrasound-Assisted Extraction of Phenolic Compounds in Morus alba Leaves

To extract the phenolic compounds from *Morus alba* leaves an ultrasonic bath (Bandelin, Sonorex, RK52, Berlin, Germany) was used, which operates at a frequency of 35 kHz. Powdered *Morus alba* leaves was placed with 10 mL of solvent extraction using the experimental conditions of the model. After centrifugations for 10 min at 1000 g, the solvent was evaporated by Buchi R-205 rotavapor and reconstituted in 2 mL of methanol/water (1:1, *v*/*v*). Finally, the extracts were filtered before the analysis using a 0.2 μm nylon syringe filter.

### 2.5. Antioxidant Capacity

The determination of antioxidant activities of *Morus alba* leaf extracts was carried out by three different assays. The results were expressed as mg Trolox equivalent/g of dry weight leaves. Three replicates of each sample were processed.

#### 2.5.1. DPPH Radical Scavenging

The protocol of Brand-Williams et al., 1995 [19] was used to develop the DPPH assay. Briefly, 0.1 mL of the extract was added to 2.9 mL of 100 µM DPPH solution in MeOH/H_2_O 1/1 (*v*/*v*) and the absorbance was determined after 30 min at 517 nm (25 °C).

#### 2.5.2. ABTS Cation Radical Scavenging

The ABTS assay was undertaken according to Re et al., 1999 [20]. ABTS radical cation (ABTS^+^) was added to EtOH to reach an absorbance of 0.7 ± 0.02 at 734 nm and 30 °C. After that, 10 µL of extract was added to 1 mL of ABTS reagent and its absorbance was measurement after 10 min.

#### 2.5.3. Ferric Reducing Antioxidant Power (FRAP)

This assay was done following the process described by Pulido et al., 2000 [21]. Under this procedure, 30 µL of the extracts was added of 0.9 mL of water and 0.9 mL of FRAP reagent. The absorbance measurement at 595 nm was undertaken after 30 min.

### 2.6. Analysis of Phenolic Compounds in Morus alba Leaf Extracts by HPLC-ESI-TOF-MS

The analysis of *Morus alba* leaf extracts was performed by HPLC-ESI-TOF-MS as previously reported by Verni et al., 2020 [22]. Three replicates of each sample were processed. The equipment consists of a UPLC system ACQUITY (Waters Corporation, Milford, MA, USA) coupled to a time-of-flight analyzer (TOF) (Waters Corporation, Milford, MA, USA). The phenolic compounds were separated using a BEH Shield RP18 column (1.7 µm, 2.1 mm × 100 mm; Waters Corporation, Milford, MA, USA). The analysis was carried out at 40 °C and the data were processed using MassLynx 4.1 software (Waters Corporation, Milford, MA, USA). 

## 3. Results and Discussion

### 3.1. Fitting the Model

Table 2 shows the values obtained for each variable response in experimental extraction conditions, according to the Box–Behnken design. 

The model was assessed in accordance with the regression significance coefficients, quadratic correlation coefficients (R^2^), quadratic correlation coefficients adjusted (R^2^ adjusted), coefficient of variation (CV) and lack of fit (Table 2). The level of significance established was α < 0.1 in accordance with previous studies [23,24]. The significant variables on the response of SPC were the linear effect of ethanol/water % (*v*/*v*) (X_1_) (*p* = 0.000899) and its quadratic effect (X_11_) (*p* = 0.000332), linear effect of solvent-to solid ratio (X_2_) (*p* = 0.002941) and its quadratic effect (X_22_) (*p* = 0.009983), the linear effect of time (X_3_) (*p* = 0.025838) and its quadratic effect (X_33_) (*p* = 0.022689) and the cross effect between ethanol/water % (*v*/*v*) with solvent-to-solid ratio (*v*/*w*) (X_12_) (*p* = 0.012855), the cross effect between ethanol/water % (*v*/*v*) with time (X_13_) (*p* = 0.032829) and the cross effect between ratio and time (X_23_) (*p* = 0.079367). The significant variables on the variable response of DPPH were the linear effect of ethanol/water % (*v*/*v*) (X_1_) (*p* = 0.003672) and its quadratic effect (X_11_) (*p* = 0.002050), linear effect of solvent-to solid ratio (*v*/*w*) (X_2_) (*p* = 0.022121) and its quadratic effect (X_22_) (*p* = 0.034571), the linear effect of time (X_3_) (*p* = 0.089993) and its quadratic effect (X_33_) (*p* = 0.071824). In addition, the significant effects on the response of ABTS were the following: ethanol/water % (*v*/*v*) (X_1_) (*p* = 0.002055) and its quadratic effect (X_11_) (*p* = 0.001100), linear effect of solvent-to solid ratio (*v*/*w*) (*p* = 0.017817) and its quadratic effect (X_22_) (*p* = 0.035413), the linear effect of time (X_3_) (*p* = 0.049034) and its quadratic effect (X_33_) (*p* = 0.023673), the cross effect between ethanol/water % (*v*/*v*) and time (X_13_) (*p* = 0.068455). Finally, the significant effects on the response FRAP were ethanol/water % (*v*/*v*) (X_1_) (*p* = 0.014110) and its quadratic effect (X_11_) (*p* = 0.004760), linear effect of solvent-to solid ratio (*v*/*w*) (X_2_) (*p* = 0.028083) and its quadratic effect (X_22_) (*p* = 0.065024), the linear effect of time (X_3_) (*p* = 0.095433) and its quadratic effect (X_33_ = 0.043803).

Statistical significance was set at the 95% of confidence level to establish all the effects. A high correlation between independent and dependent factors was obtained with quadratic correlation coefficient (R^2^) from 92.17–99.48%, which, with the exception of the FRAP, provided a good correlation but lower than the other ones (R^2^ = 86.89%). According to a previous study, R^2^ should be at least 0.80 for a good fit [25]. In addition, the verification of the suitability of the model was carried out according to the *p*-value obtained, it being non-significant (*p* > 0.05) means that the model fits well (Table 3). Moreover, as the *p*-value was lower than 0.05 for all cases, all models were considered statistically acceptable.

### 3.2. Response Surfaces Methodology Analysis

**Figure 1:**
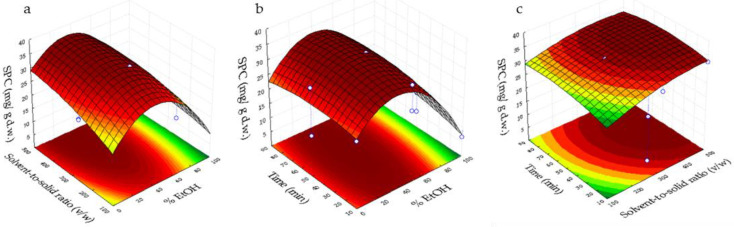
Response surfaces of combined effects for sum of phenolic compounds (SPC). (**a**) Solvent-to-solid ratio (*v*/*w*) vs. % EtOH; (**b**) Time vs. %EtOH; and (**c**) time vs. solvent-to-solid ratio (*v*/*w*).

**Figure 2:**
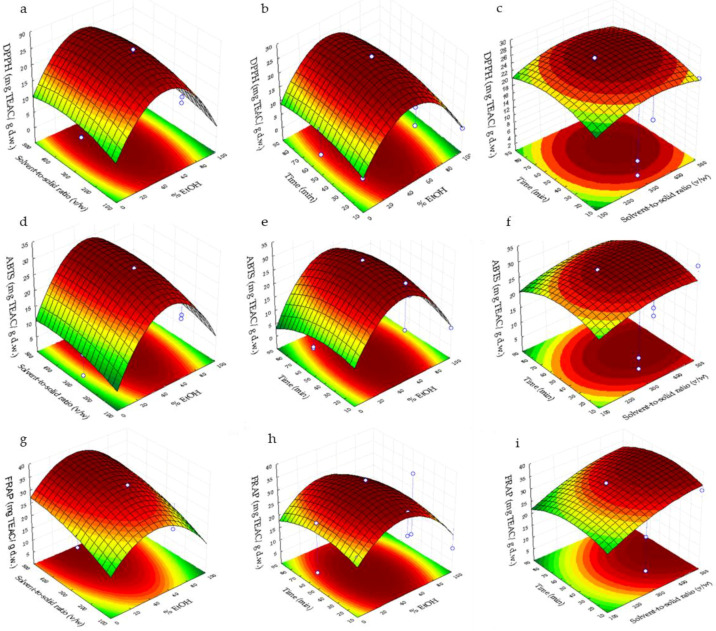
Response surface plots showing combined effects for DPPH, ABTS and FRAP assay. (**a**,**d**,**g**) Solvent-to-solid ratio (*v*/*w*) vs. % EtOH; (**b**,**e**,**h**) time vs. %EtOH; and (**c**,**f**,**i**) time vs. solvent-to-solid ratio (*v*/*w*).

Figure 1 and Figure 2 plot the three-dimensional response surfaces, which show the effects of % EtOH (X_1_) with solvent-to-solid ratio (*v*/*w*) (X_2_) (a, d, g), %EtOH (X_1_) with time (min) (X_3_) (b, e, h) and time (X_3_) with solvent-to-solid ratio (*v*/*w*) (X_2_) (c, f, i) on the SPC, DPPH, ABTS and FRAP.

Analyzing Figure 1a, the highest SPC was in the range of 20–50% ethanol/water and 300–500 of solvent-to-solid ratio (*v*/*w*), whereas the maximum concentration of SPC in Figure 1b was observed at 20–50% ethanol/water and 15–90 min. Finally, in Figure 1c the highest value was obtained at 30–80 min and 400–500 of solvent-to-solid ratio (*v*/*w*). The maximum value of the sum of phenolic compounds could be explained as a result of the positive influence of the quadratic effect of EtOH and linear effect of the solvent-to-solid ratio. In addition, the decrease of this response could be mainly due to the linear negative effect of EtOH.

In respect of DPPH, its maximum content was obtained in a range of 30–60% ethanol/water at 150–500 of solvent-to-solid ratio (Figure 2a), whereas the highest DDPH value shows in the range of 30–70 min and 40–60% ethanol/water in Figure 2b and 250–400 of solvent-to-solid ratio (*v*/*w*) and 35–70 min in Figure 2c. The increase in the DPPH response could be due to the positive quadratic effect of EtOH as this variable exerts the highest effect on this response in comparison with the rest of the variables. In addition, with regard to ABTS response, its maximum value was shown between 40–60 % ethanol/water at 200–500 of solvent-to-solid ratio (*v*/*w*) (Figure 2d), 25–65 min and 40–60% ethanol/water in Figure 2e and 250–500 of solvent-to-solid ratio (*v*/*w*) at 25–65 min in Figure 2f. The quadratic of EtOH and linear solvent-to-solid ratio were the variables which provide a higher effect on the ABTS response, which could explain the increase in this response. Finally, the highest value of FRAP can be observed at 40–60% of ethanol/water and 450–500 of solvent-to-solid ratio (*v*/*w*) (Figure 2g), 30–55 min and 40–55% of ethanol/water (Figure 2h), whereas the maximum content of FRAP in Figure 2i was obtained 400–500 of solvent-to-solid ratio (*v*/*w*) and 25–55 min. Variables which exert the highest effect on this response were the quadratic of EtOH and solvent-to-solid ratio, which explain the maximum value obtained in FRAP response. 

### 3.3. Optimization of Ultrasonic-Assisted Extraction 

#### 3.3.1. Optimal Ultrasonic-Assisted Extraction Conditions 

After the analysis of the 3-D plots and the choice of the optimal conditions, accuracy of the mathematical model was established comparing the predicted and experimental data.

Table 3 shows the results of the sum phenolic compounds and in vitro antioxidant activity by the three different assays from *Morus alba* leaf extract obtained at optimum conditions. The same following optimal conditions were established for all responses: 40% ethanol/water, 35 min and 400 of solvent-to-solid ratio (*v*/*w*), obtaining predictable values of 36 ± 2 mg/g d.w. for the sum of phenolic compounds, 25 ± 3, 29 ± 3, 36 ± 4 mg TEAC/ g d.w. for DPPH, ABTS and FRAP. According to the results, the extraction time was lower than that reported by a previous study, whose extraction conditions in *Morus alba* leaves were methanol (each 2 L) for 4 h at 60 °C obtaining 23.2 and 55.4 mg gallic acid equivalent/g d.w. [26]. In addition, another study reported an extraction of phenolic compounds from *Morus alba* leaves with 80% aqueous methanol acidified with formic acid (1%) sonicated at 25 ± 5 °C for 60 min [27]. However, Kim et al., 2020 [13] reported a similar extraction time of 30 min in *Morus* samples using 70% methanol with the ultrasonic extractor. Another study reported an ultrasonic-assisted extraction by using methanol/water mixture at a shorter extraction time than in the present study (10 min) and ¼ of solid-to-solvent ratio (*w*/*v*) to obtain 0.31 mg GAE/g d.w. and 0.19 mg TEAC/g d.w. for total phenolic compounds and DPPH [12]. Nevertheless, this DPPH value was 99% lower than that obtained by the present study [12]. Another study reported a similar concentration range for the sum of phenolic compounds 19.17–58.47 mg/g obtained by pressurized liquid extraction in *Morus alba* leaves using a similar mixture EtOH/H_2_O 50:50 (*v:v*) as solvent, at 200 °C for 20 min in static cycle [18]. Therefore, it has been proven that ultrasonic-assisted extraction at optimum conditions is a process as efficient in the phenolic recovery from *Morus alba* leaves as pressurized liquid extraction. Therefore, the application of ultrasonic-assisted extraction could be an efficient alternative to other green techniques reducing the cost of extraction operations. Nevertheless, there is a wide variability among the phenolic contents and antioxidant activities in mulberry leaves due to the different cultivars of *Morus alba* leaves, different extraction techniques used and different analytical methods used in each studies [12].

#### 3.3.2. Determination of Phenolic Compounds in *Morus alba* Leaf Extracts by HPLC-MS 

Phenolic compounds of *Morus alba* leaf extract were identified by HPLC-ESI-TOF-MS according to their mass data and by comparing them with literature, the co-elution with commercial standards (when possible) and with several databases. Mass data, experimental and calculated *m*/*z*, error and Fit Conf %, mainly in source fragments and molecular formulae (M-H)^−^, were considered.

As reported in Table 4, 21 phenolic compounds were detected in the *Morus alba* leaf extracts, including seven phenolic acid derivatives and fourteen flavonols. Supplementary Figure S1 shows the base peak chromatogram obtained by HPLC-ESI-TOF-MS for each compound in the *Morus alba* leaf extract obtained at optimal ultrasonic-assisted extraction conditions. Peak 1 at 2.02 min and *m*/*z* 315.0714 showed a molecular formula of C_13_H_15_O_9_ and fragment ions at *m*/*z* 153.0162 and 109.0279; according to a previous study [18] it was assigned to protocatechuic acid-glucoside. Peak 2 at 3.78 min with *m*/*z* 353.0870 with a molecular formula of C_16_H_17_O_9_ and fragments at *m*/*z* 179.0336, 191.0551, 135.0436 and 173.0419 was identified as 3-caffeoylquinic acid, which has been identified previously in *Morus alba* leaf extracts [12,18,28,29]. Peak 3 at 5.32 min with *m*/*z* 515.1406 and fragments at *m*/*z* 341.0868, 191.0472, 179.0314 with a molecular formula of C_22_H_27_O_14_, was proposed to be chlorogenic acid hexose [18]. Peak 4 at 5.52 min with *m*/*z* 353.0866 with a molecular formula of C_16_H_17_O_9_ and fragment ions at *m*/*z* 191.0551, 173.0455 and 135.0436, this compound was proposed to be 5-caffeoylquinic acid (chlorogenic acid) [28]. Peak 5 at 5.75 min with *m*/*z* 353.0873 and with a molecular formula of C_16_H_17_O_9_ presented a fragment ion at *m*/*z* 191.0553, 173.0432 and 179.0428, which correspond with 4-caffeoylquinic acid (cryptochlorogenic acid) [28]. Peak 6 at 6.05 min with *m*/*z* 771.1996 and fragment ions with *m*/*z* 609.1451, 463.0819, 299.0175 and 300.0279 and a molecular formula of C_33_H_39_O_21_ was proposed as quercetin rhammosyl hexoside, which has been identified in mulberry samples [30]. Peak 7 with *m*/*z* 625.1411 and fragment ions at *m*/*z* 300.0234 and 301.0336 with a molecular formula of C_27_H_29_O_17_ correspond with quercetin di-hexoside, which has been previously identified in mulberry fruit and leaves [18,30]. At 7.35 min (peak 8) with *m*/*z* 609.1456 and fragment ions *m*/*z* 285.0388 and 447.0918 with C_27_H_29_O_16_ was identified as kaempferol-hexoside-hexoside, which has been identified previously in mulberry leaves [27]. Peak 9 at 7.47 min with a *m*/*z* 711.1434 and a fragment ion *m*/*z* 667.1544 with C_30_H_31_O_20_ was proposed to be quercetin malonyl di-hexoside, which has been identified previously in white and black mulberry leaf extracts [27]. Peak 10 at 8.37 min with *m*/*z* 695.1463 and ion fragments 651.1573, 489.1035, 531.1118 was detected as kaempferol-malonyl-dihexoside [27]. Peak 11 at 9.64 min with *m*/*z* 755.2035 with fragment ions *m*/*z* at 300.0264 and 271.0244 and a molecular formula of C_33_H_39_O_20_ was detected as kaempferol rutinoside hexoside, which has been identified in *Morus alba* leaf extract [18,31]. Peaks 12 and 13 (10.07 and 10.32 min) with a molecular formula of C_27_H_29_O_16_ and *m*/*z* 609.1456 and as fragment ion *m*/*z* 301.03 correspond with isomers of rutin [18,28]. Peak 14 at 10.53 min with *m*/*z* 463.0894 with a molecular formula of C_21_H_19_O_12_ and fragment ions *m*/*z* 255.0298 and 300.0277 was detected as isoquercitrin (quercetin-3-glucoside) [28]. Peak 15 (10.79 min) with *m*/*z* 593.1511 and fragment ions *m*/*z* 285.0381 with a molecular formula of C_27_H_29_O_15_ was detected as kaempferol-3-rutinoside [28]. Peak 16 (11.2 min) with *m*/*z* 593.1519 and fragment ions *m*/*z* 353.0872 and 473.2368 with a molecular formula of C_27_H_29_O_15_ was detected as vicenin-2 [28]. Peaks 17, 19 and 20 (11.41, 11.89 and 12.1 min) with *m*/*z* 505.0984 with a molecular formula C_23_H_21_O_13_ and fragments *m*/*z* 255, 271, 300 and 301 were proposed to be isomers of quercetin-3-O-(6-acetylglucoside) [18]. Peak 18 at 11.51 min with a molecular formula of C_21_H_19_O_11_ and *m*/*z* 447.0916 with a fragment ion *m*/*z* 284.0318 was proposed to be kaempferol 3-o-glucoside [28]. Peak 21 at 12.48 min with a *m*/*z* 489.1051 (C_23_H_21_O_12_) and a ion fragments *m*/*z* 285.0398 and 191.0552 was detected as kaempferol-3-O-6″-O-acetyl-β-D-glucopyranoside [27,28]. 

Twenty-one phenolic compounds were also quantified. The quantification of phenolic compounds in *Morus Alba* leaf optimum extracts was done by using the calibration curves of standards. A good linearity was obtained in all calibration curves (r^2^ > 0.9954). The limit of detection (LOD) and limit of quantification (LOQ) were 0.04–0.47 mg/L, and 0.14–1.57 mg/L, respectively.

The quantitative results of phenolic compounds from the two *M. alba* leaf extracts by HPLC-MS are shown in Table 5. According to the results, phenolic acid derivatives were the most abundant phenolic compounds in *Morus alba* leaf optimum extract. These results are in agreement with previous studies [18,32]. Protocatechuic acid-glucoside was the most concentrated phenolic acid derivative, followed by 4-caffeoylquinic acid (cryptochlorogenic acid), which represent more than 33% of total phenolic acid derivatives. These results are similar to those obtained by a previous study, which reported cryptochorogenic acid as the most concentrated phenolic compound in a range of 4.6–16.5 mg/g d.w. in different *Morus alba* leaves genotypes [18]. Another study reported a similar concentration of chlorogenic acid in *Morus alba* leaf extract (1.7–2.3 mg/g d.w.) [33]. The total content of caffeoylquinic acids was in the same order of magnitude as that obtained by a previous study in white and black mulberry leaves (6.43–10.05 mg/g d.w.) [32]. In addition, the most concentrated flavonol was quercetin-3-O-(6-acetylglucoside) followed by rutin isomer b. These results are in concordance with previous studies that reported rutin as the most abundant flavonol in a different genotype of *Morus alba* leaves, whose content was in a similar order of magnitude (0.58–2.98 mg/g d.w.) than that obtained in the present study [18,32]. Another study reported a similar content of rutin obtained in *Morus alba* leaf collected from different regions (3.10 mg/g d.w.) [26]. The total flavonols content was 5.2 ± 0.8 mg/g d.w., which was in a similar order of magnitude as that reported by Sanchez-Salcedo et al., 2015 in different white and black *Morus alba* leaves (3.66–9.75 mg/g d.w.) [32]. In addition, the total phenolic content in *Morus alba* leaf extract was 16.4 ± 0.6 mg/g d.w., which is in a similar order of magnitude as that provided by a previous study in different *Morus alba* leaves genotypes (19.171–58.474 mg/g d.w.) [18]. Therefore, the difference in the phenolic content obtained in the present study in comparison with previous research may be caused by the different climatic conditions and environmental conditions (temperature, altitude, soil, cultivar, humidity) [12,34].

## 4. Conclusions

In conclusion, an optimization of an ultrasonic-assisted extraction parameters (percentage of ethanol, solvent/sample ratio (*v*/*w*) and extraction time) for the phenolic recovery of *Morus alba* leaves was established for the first time by using a mathematical model. This is an important step in assessing the quality control of mulberry leaves. The highest amounts of phenolic compounds and the correspondent antioxidant activity evaluated by DPPH, ABTS and FRAP were obtained at 40% ethanol (water solution), 35 min and a solvent/sample ratio (*v*/*w*) of 400. Among phenolic compounds, 21 compounds were identified by HPLC-ESI-TOF-MS and the most concentrated were protocatechuic acid-glucoside, cryptochlorogenic acid, quercetin-3-O-(6-acetylglucoside) and rutin, which represent more than 55% of the total phenolic content. As demonstrated, *Morus alba* leaf extracts obtained in these optimum conditions reported high variability on phenolic content, thus, the proposed method helps the quality control of the *Morus alba* leaves for nutra-pharmaceutical purposes.

## Figures and Tables

**Table 1:** Values for the dependent factors, and response variables obtained in the Box-Behnken design (BBD).

Run	Dependent Factors			Response Variables			
	**X_1_**	**X_2_**	**X_3_**	**SPC**	**DPPH**	**ABTS**	**FRAP**
1	100	500	50	12.17	3.38	9.05	30.41
2	0	20	50	14.08	2.84	5.92	6.87
3	50	500	90	31.40	22.35	27.32	27.39
4	0	500	50	30.07	12.29	6.39	26.63
5	50	500	10	32.65	22.21	31.29	32.54
6	50	260	50	32.73	24.60	29.08	32.58
7	50	260	50	33.04	27.55	27.76	35.34
8	100	260	90	12.69	5.89	8.12	13.30
9	0	260	90	23.23	7.93	3.40	19.37
10	0	260	10	22.85	7.89	10.30	23.67
11	100	20	50	5.37	1.84	10.06	5.38
12	50	20	90	26.44	17.00	15.20	20.69
13	50	20	10	24.18	15.67	14.39	23.41
14	100	260	10	6.65	3.89	6.69	9.79
15	50	260	50	33.76	25.95	27.51	32.24

X_1_: Ethanol/water ratio (*v*/*v*), X_2_: solvent/sample ratio (*v*/*w*) and X_3_: extraction time (min). The sum of phenolic compounds (SPC) was given in mg/g d.w. DPPH, ABTS and FRAP were expressed as mg Trolox eq./g d.w.

**Table 2:** Coefficients of regression, effects and analysis of variance (ANOVA) of the model for the response variables.

	SPC		DPPH		ABTS		FRAP	
	**Coefficients**	**Effects**	**Coefficients**	**Effects**	**Coefficients**	**Effects**	**Coefficients**	**Effects**
β_0_	10.85230 *	20.1500	−3.42399	10.26411	−1.56191	12.34545	8.764200 *	19.95608
Linear								
β_1_	0.48233 *	−13.3355	0.66784 *	−3.98835	0.69638 *	1.97903	0.389353 *	−4.41331
β_2_	0.05756 *	9.0560	0.05799 *	5.72199	0.05052 *	7.12160	0.059015 *	15.15499
β_3_	0.12328 *	1.8605	0.17588 **	0.87715	0.19197 *	−2.15594	0.195844 **	−2.16710
Cross product								
β_12_	−0.00019 *	−4.5953	−0.00016	−3.95706	−0.00003	−0.73700	0.000110	2.63046
β_13_	0.00071 *	2.8316	0.00025	0.98140	0.00104 *	4.16383	0.000977	3.90610
β_23_	−0.00009 **	−1.7544	−0.00003	−0.58807	−0.00012	−2.38837	−0.000063	−1.21595
Quadratic								
β_11_	−0.00601 *	15.0324	−0.00677 *	16.92810	−0.00721 *	18.01628	−0.005108 *	12.77022
β_22_	−0.00005 *	2.7197	−0.00007 *	4.02061	−0.00005 *	3.09327	−0.000057 **	3.29606
β_33_	−0.00112 *	1.7866	−0.00169 **	2.70756	−0.00239 *	3.81799	−0.002553 *	4.08468
R^2^	0.98932 0.054291		0.99476 0.461657		0.92168 0.052922		0.86893 0.083124	
*p* (Lack of fit)								

* Significant at *p* < 0.05 level, ** Significant at *p* < 0.1 level.

**Table 3:** Optimal conditions for ultrasonic-assisted extraction.

Optimal Conditions	SPC	DPPH	ABTS	FRAP
Ethanol/water % (*v*/*v*)	40	40	40	40
Solvent-to-solid ratio (*v*/*w*)	400	400	400	400
Time (min)	35	35	35	35
Predicted	36 ± 2	25 ± 3	29 ± 3	36 ± 4
Observed	37.3 ± 0.7	27.6 ± 0.9	30.5 ± 0.3	36.8 ± 0.2
Significant differences	N.S.	N.S.	N.S.	N.S.

N.S.: no significant differences. SPC was expressed as mg/g d.w. DPPH, ABTS and FRAP were expressed as mg trolox/g sample d.w.

**Table 4:** Table of identification of phenolic compounds from optimum *Morus alba* leaf extract by HPLC-MS.

Peak	RT	*m/z* Experimental	*m/z* Calculated	Tolerance (ppm)	Error (ppm)	Fit Conf %	In Source Fragments	Molecular Formula	Compound
**1**	2.02	315.0714	315.0716	10	1.3	99.96	153.0162, 109.0279	C_13_H_15_O_9_	Protocatechuic acid-glucoside
**2**	3.78	353.087	353.0873	10	−0.8	99.98	179.0336, 191.0551, 135.0436, 173.0419	C_16_H_17_O_9_	3-Caffeoylquinic acid (neochlorogenic acid)
**3**	5.32	515.1405	515.1401	10	0.8	99.85	341.0868, 191.0472, 179.0314	C_22_H_27_O_14_	Chlorogenic acid hexoside
**4**	5.52	353.0866	353.0873	10	−2	99.96	191.0551, 179.0337, 173.0455	C_16_H_17_O_9_	5-caffeoylquinic acid (chlorogenic acid)
**5**	5.75	353.0873	353.0873	10	0.0	96.51	191.0553 173.0432 179.0428	C_16_H_17_O_9_	4-Caffeoylquinic acid (cryptochlorogenic acid)
**6**	6.05	771.1996	771.1984	10	1.6	98.28	609.1451, 463.0819, 300.0279 299.0175	C_33_H_39_O_21_	Quercetin rhammosyl hexoside
**7**	6.64	625.1411	625.1405	10	1	99.49	300.0234, 301.0336	C_27_H_29_O_17_	Quercetin dihexoside
**8**	7.35	609.1446	609.1456	10	−1.6	94.92	285.0388, 447.0918	C_27_H_29_O_16_	Kaempferol-hexoside-hexoside
**9**	7.47	711.1434	711.1409	10	3.5	98.63	667.1544	C_30_H_31_O_20_	Quercetin malonyl di-hexoside
**10**	8.37	695.1463	695.146	10	0.4	98.62	651.1573, 489.1035, 531.1118	C_30_H_31_O_19_	Kaempferol-malonyl-dihexoside
**11**	9.64	755.2037	755.2035	10	0.3	99.55	300.0264, 271.0244	C_33_H_39_O_20_	Kaempferol rutinoside hexoside
**12**	10.07	609.1467	609.1456	10	1.8	99.32	301.0321	C_27_H_29_O_16_	Rutin isomer a
**13**	10.32	609.1483	609.1456	10	4.4	84.77	301.0343	C_27_H_29_O_16_	Rutin isomer b
**14**	10.53	463.0894	463.0877	10	3.7	94.13	255.0298, 300.0277	C_21_H_19_O_12_	Isoquercitrin (Quercetin-3-glucoside)
**15**	10.79	593.1511	593.1506	10	0.8	99.78	285.0381	C_27_H_29_O_15_	Kaempferol-3-rutinoside
**16**	11.20	593.1519	593.1506	10	2.2	99.99	353.0872, 473.2368	C_27_H_29_O_15_	Vicenin-2
**17**	11.41	505.0984	505.0982	10	0.4	99.71	255.0289, 271.0237, 300.0265 301.0332	C_23_H_21_O_13_	Quercetin-3-O-(6-acetylglucoside) isomer a
**18**	11.51	447.0916	447.0927	10	−2.5	88.67	284.0318	C_21_H_19_O_11_	Kaempferol 3-o-glucoside
**19**	11.89	505.0967	505.0982	10	−3	99.96	255.0277, 271.0230, 301.0303, 300.0256	C_23_H_21_O_13_	Quercetin-3-O-(6-acetylglucoside) isomer b
**20**	12.11	505.0983	505.0982	10	0.2	98.9	255.0367, 271.0314, 300.0284, 301.0421	C_23_H_21_O_13_	Quercetin-3-O-(6-acetylglucoside) isomer c
**21**	12.48	489.1051	489.1033	10	3.7	87.34	285.0398, 191.0552	C_23_H_21_O_12_	Kaempferol-3-O-6″-O-acetyl-β-D-glucopyranoside

**Table 5:** Quantification of phenolic compounds in the two cultivars of *Morus alba* leaves (MAL1 and MAL2) by HPLC-MS expressed as mg/g d.w and antioxidant activity expressed as mg Trolox/g d.w.

Compound	MAL1	MAL2
Protocatechuic acid-glucoside	9.3 ± 0.3	4.1 ± 0.4
3-Caffeoylquinic acid (neochlorogenic acid)	3.4 ± 0.1	1.5 ± 0.07
Chlorogenic acid hexoside	0.95 ± 0.05	0.4 ± 0.02
5-caffeoylquinic acid (chlorogenic acid)	3.4 ± 0.2	1.5 ± 0.2
4-Caffeoylquinic acid (cryptochlorogenic acid)	8.4 ± 0.7	3.7 ± 0.2
Quercetin rhammosyl hexoside	0.08 ± 0.01	0.037 ± 0.008
Quercetin dihexoside	0.61 ± 0.05	0.27 ± 0.03
Kaempferol-hexoside-hexoside	0.57 ± 0.03	0.25 ± 0.02
Quercetin malonyl di-hexoside	0.11 ± 0.01	0.05 ± 0.02
Kaempferol-malonyl-dihexoside	0.022 ± 0.002	0.010 ± 0.001
Kaempferol rutinoside hexoside	0.29 ± 0.03	0.13 ± 0.01
Rutin isomer a	0.81 ± 0.01	0.36 ± 0.04
Rutin isomer b	1.8 ± 0.1	0.8 ± 0.1
Isoquercitrin (Quercetin-3-glucoside)	1.8 ± 0.2	0.79 ± 0.06
Kaempferol-3-rutinoside	0.25 ± 0.03	0.11 ± 0.03
Vicenin-2	0.41 ± 0.05	0.18 ± 0.02
**Quercetin-3-O-(6-acetylglucoside) isomer a**	2.44 ± 0.06	1.08 ± 0.05
Kaempferol-3-O-glucoside	0.20 ± 0.02	0.09 ± 0.02
**Quercetin-3-O-(6-acetylglucoside) isomer b**	0.16 ± 0.01	0.07 ± 0.04
**Quercetin-3-O-(6-acetylglucoside) isomer c**	0.072 ± 0.004	0.03 ± 0.01
**Kaempferol-3-O-6** **″** **-O-acetyl-β-D-glucopyranoside**	2.3 ± 0.2	1.0 ± 0.3
Sum flavonols	11.9 ± 0.3	5.2 ± 0.8
Sum phenolic acid derivatives	25.4 ± 0.5	11.2 ± 0.8
Sum of phenolic compounds (SPC)	37.3 ± 0.7	16.4 ± 0.6
DPPH	27.6 ± 0.9	16.4 ± 0.4
ABTS	30.5 ± 0.3	21.6 ± 0.1
FRAP	36.8 ± 0.2	20.1 ± 0.3

## Data Availability

Data is contained within the article and Supplementary Material.

## Supplementary Materials

The following supporting information can be downloaded at: https://www.mdpi.com/article/10.3390/foods11030314/s1, Figure S1: Base peak chromatogram (BPC) obtained from HPLC-ESI-TOF-MS analysis of *Morus alba* leaf extract obtained by optimal ultrasonic-assisted extraction conditions. Peaks have been numbered according to the elution order.
