# Supplementary material for: The Establishment of Ultrasonic-Assisted Extraction for the Recovery of Phenolic Compounds and Evaluation of Their Antioxidant Activity from Morus alba Leaves
Source: Foods. 2022 Jan 24;11(3):314. doi: 10.3390/foods11030314 (PMC8834592; doi:10.3390/foods11030314)
Supplement: Supplementary file 1 [file foods-11-00314-s001.zip › foods-1564554-supplementary.pdf]

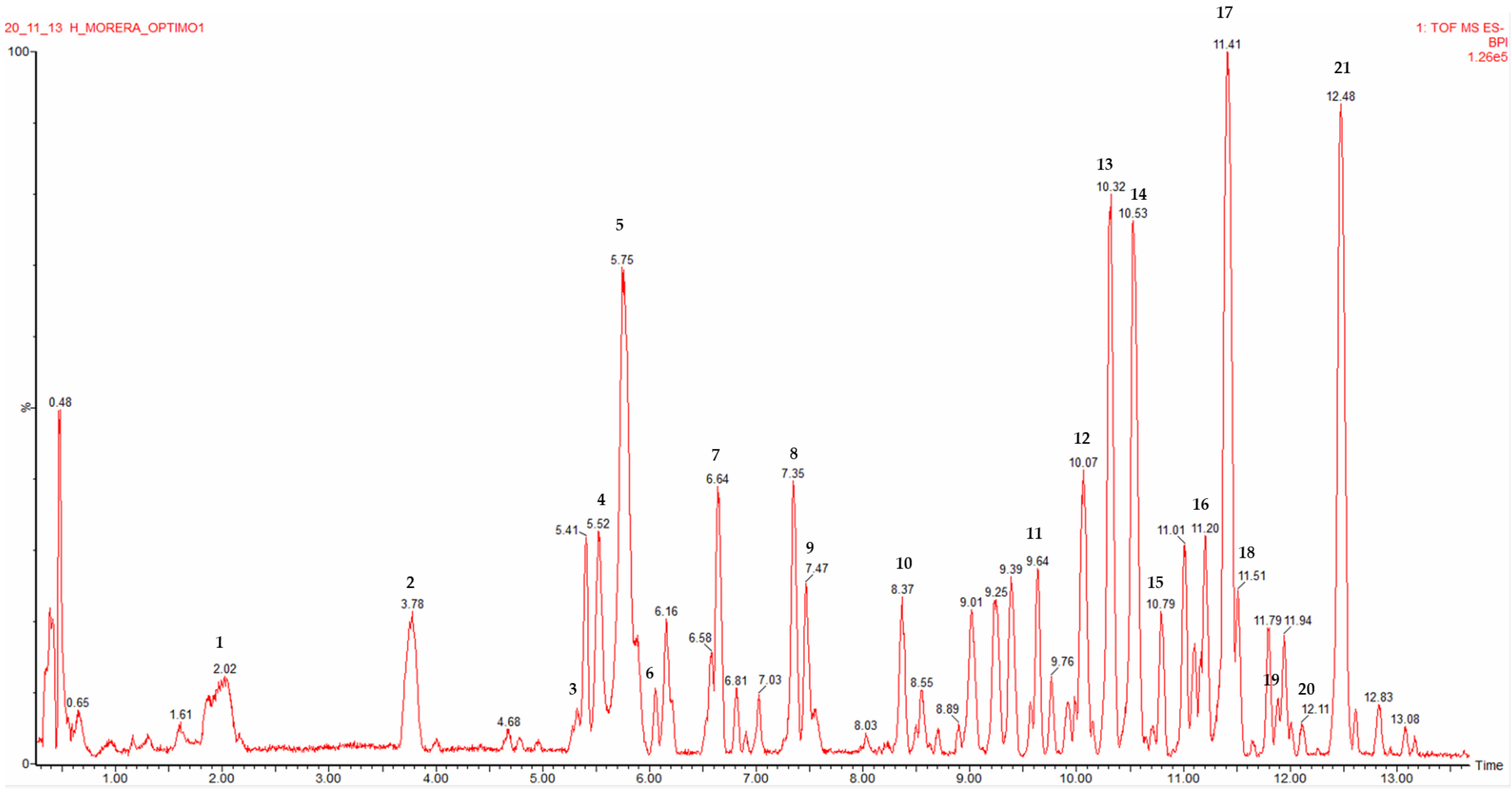

Figure S1. Base peak chromatogram (BPC) obtained from HPLC-ESI-TOF-MS analysis of *Morus alba* leaf extract obtained by optimal ultrasonic assisted extraction conditions. Peaks have been numbered according to the elution order.
